# Multilingual RECIST classification of radiology reports using supervised learning

**DOI:** 10.3389/fdgth.2023.1195017

**Published:** 2023-06-14

**Authors:** Luc Mottin, Jean-Philippe Goldman, Christoph Jäggli, Rita Achermann, Julien Gobeill, Julien Knafou, Julien Ehrsam, Alexandre Wicky, Camille L. Gérard, Tanja Schwenk, Mélinda Charrier, Petros Tsantoulis, Christian Lovis, Alexander Leichtle, Michael K. Kiessling, Olivier Michielin, Sylvain Pradervand, Vasiliki Foufi, Patrick Ruch

**Affiliations:** ^1^ HES-SO\HEG Genève, Information Sciences, Geneva, Switzerland; ^2^ SIB Text Mining Group, Swiss Institute of Bioinformatics, Geneva, Switzerland; ^3^ Division of Medical Information Sciences, University Hospitals of Geneva, Geneva, Switzerland; ^4^ Inselspital – Bern University Hospital and University of Bern, Bern, Switzerland; ^5^ Department of Radiology, Clinic of Radiology & Nuclear Medicine, University Hospital Basel, University of Basel, Basel, Switzerland; ^6^ Department of Radiology and Medical Informatics, University of Geneva, Geneva, Switzerland; ^7^ Precision Oncology Center, Oncology Department, Centre Hospitalier Universitaire Vaudois – CHUV, Lausanne, Switzerland; ^8^ Department of Oncology, Kantonsspital Aarau, Aarau, Switzerland; ^9^ Department of Medical Oncology and Hematology, University Hospital Zurich, Zurich, Switzerland

**Keywords:** supervised machine learning, narrative text classification, RECIST, radiology reports, language models

## Abstract

**Objectives:**

The objective of this study is the exploration of Artificial Intelligence and Natural Language Processing techniques to support the automatic assignment of the four Response Evaluation Criteria in Solid Tumors (RECIST) scales based on radiology reports. We also aim at evaluating how languages and institutional specificities of Swiss teaching hospitals are likely to affect the quality of the classification in French and German languages.

**Methods:**

In our approach, 7 machine learning methods were evaluated to establish a strong baseline. Then, robust models were built, fine-tuned according to the language (French and German), and compared with the expert annotation.

**Results:**

The best strategies yield average F1-scores of 90% and 86% respectively for the 2-classes (Progressive/Non-progressive) and the 4-classes (Progressive Disease, Stable Disease, Partial Response, Complete Response) RECIST classification tasks.

**Conclusions:**

These results are competitive with the manual labeling as measured by Matthew's correlation coefficient and Cohen's Kappa (79% and 76%). On this basis, we confirm the capacity of specific models to generalize on new unseen data and we assess the impact of using Pre-trained Language Models (PLMs) on the accuracy of the classifiers.

## 1. Introduction

Personalized Oncology aims at providing treatments precisely adapted to the tumor and the genetic characteristics of the patient. To improve clinical care in this area, the five Swiss Universities hospitals decided to join forces within the Swiss Personalized Oncology (SPO) program of the Swiss Personalized Health Network (SPHN) in order to share data and develop new decision-support instruments. As part of this, we report on our efforts to automatically extract Response Evaluation Criteria in Solid Tumors (RECIST) categories out of radiology reports using machine learning based approaches.

The version 1.1 of RECIST allows the expert to classify a tumor response using four distinct categories (1, 2), namely Complete Response (CR), Partial Response (PR), Stable Disease (SD), and Progressive Disease (PD). Unlike clinical trials where the treatment response is a primary measurement, real world radiology reports are weakly formatted interpretations of an imaging study based on the observation of the radiologist (e.g. see Hersh et al. (3) for a collection of radiology reports). Mostly written in a narrative style, this document may be split into multiple sections (e.g., “clinical information”, “conclusion”, etc.), and will be used by the physician to monitor the evolution of a tumor on treatment. In addition to the free-text section, an assessment relying on RECIST criteria can occasionally be embedded into the radiology report, but this is never a routine practice.

This lack of standardization involves a considerable time investment for the oncologists to retrieve information and limits the interoperability of data across different health centers. Thus, Natural Language Processing (NLP) approaches can opportunely be used to support both clinicians and researchers by analyzing and extracting knowledge from free texts. Well described in the literature (4–6), using machine learning (ML) methods to perform NLP tasks has already proved effective and is likely to supply reliable results in oncology-related tasks (7–9) such as the automatic classification of clinical narratives or the extraction of cancer treatment response. In this article, we are reporting on the assessment of effective strategies to automatically associate a RECIST label on existing radiological reports and to tailor them to the actual difference between Swiss hospitals (multiple languages, writing styles, etc.).

## 2. Methods

### 2.1. Data selection

In each of the five Swiss teaching hospitals, an initial dataset was built with 120 radiological reports manually selected. These reports are based on three specific imaging techniques (Computed Tomography (CT), Magnetic Resonance Imaging (MRI) and Positron Emission Tomography (PET)) and focus uniformly on six disease types (breast cancer, glioblastoma, lung cancer, melanoma, prostate cancer, and gastrointestinal cancer). More detailed information on the inclusion/exclusion criteria are specified in the SPO guidelines for treatment response extraction (see Supplementary Materials “Treatment Response Guidelines”).

Two language-oriented corpora were then produced. The corpus “GERMAN” gathers the documents written in German and originating from the University Hospital Basel (USB), the University Hospital Bern (Insel) and the University Hospital Zurich (USZ); and the corpus “FRENCH” gathers the documents written in French and originating from the Geneva University Hospitals (HUG) and the Lausanne University Hospital (CHUV).

Before any manual or automatic processing, the datasets were de-identified based on the HIPAA Privacy Rule (10). For the experiments, we focused on the conclusion sections from these reports (with 34 and 56 words on average, respectively for the French and German documents). With respect to the guidelines mentioned above, experts from the hospitals have manually classified all the documents in order to generate training and testing data for the subsequent evaluations. To that extent, we gathered at least two manual labels on each conclusion and estimated an Inter-Annotator Agreement (IAA) (see Supplementary Materials “Inter-annotator agreement evaluation”). This agreement allowed us to discard the documents with a high level of ambiguity to streamline the first stage of experiments (11).

### 2.2. Manual classification

Each data provider organized a group of annotators (up to 3 experts) who manually annotated the radiology reports by following strict guidelines. A classification was performed specifically on the conclusion section of each report in anticipation of the machine learning experiments. Based on the RECIST 1.1 standard, five classes were extracted: (1) complete response (CR), (2) partial response (PR), (3) stable disease (SD), (4) progression (PD), and (5) unknown for the reports where the author does not state any conclusion.

An additional classification subcategory called “Dissociated response” = [yes/no, default is no] was assigned in order to consider the reporting of lesions responding differently to the treatment within the same report conclusion. Moreover, an attribute labeled “Low confidence” = [yes/no, default is no] was added for cases where the report's author speculates about the response. The purpose of these two annotations is to select a subset of documents for which the conclusions are more homogenous (no mixed response, non low confidence).

In a later phase, the differences between the experts' annotations were solved with the aim of creating a gold standard corpus for each language and at each institution. Thus, the documents with no consensus were discarded from the corpora, while balancing the HUG dataset led to a set of 122 reports. THE final FRENCH and GERMAN corpora respectively contain 242 and 343 documents. Training and test sets for the automatic classification experiments are built based on these gold standard corpora (see Table 1 for the distribution of the labels).

**Table 1:** Distribution of RECIST annotations on each institutional dataset. Over the 585 annotations, “Dissociated response” is assigned to 59 documents, and “Low confidence” is assigned to 65 documents.

RECIST	CHUV	HUG	Insel	USB	USZ	Total
Complete response	12	22	7	29	21	91
Partial response	30	21	42	13	25	131
Stable disease	20	36	30	15	30	131
Progressive disease	56	40	41	31	44	212
Unknown	2	3	0	15	0	20
Total	120	122	120	103	120	585

In addition to this, one of the HUG annotators has delivered an additional judgment on the complete FRENCH corpus. This additional annotation phase aims at strengthening the IAA in order to prepare a batch of experiments comparing the behavior of different ML-based approaches in handling data from another institution.

### 2.3. Automatic classification

ML refers to a domain in Artificial Intelligence (AI) that aims at developing algorithms able to learn from training data and make decisions based on statistical probabilities (also called “predictions”). As opposed to rule-based programming, ML is able to improve by increasing and diversifying training data and these approaches have already shown good results in many scientific fields, including medicine (7–9, 12–15). Yet, there are limitations to such approaches, which must be emphasized: wrong predictions can occur because of insufficient, imbalanced, or unknown data. For each of these limitations, AI researchers have developed recovery strategies and thus radiology reports may represent a fairly good resource for automation such as the evaluation of the tumor response to a specific therapy, which would make possible the medical encoding of RECIST categories at scale.

Among the different forms of ML, we explored the opportunity to build supervised models (*i.e.,* models trained on labeled data that can be used to predict unobserved labels for new data). As mentioned above, the set of documents classified by the human experts will serve as ground truth for the learning phase. Thereby, the system processed the data and inferred rules that map specific textual elements related to the different RECIST classes, whether for a binary classification (progressive/non-progressive) or a multiclass classification (CR, PR, SD or PD).

### 2.4. Machine learning pipeline

Our ML-based pipeline includes five steps:

• Preprocessing

Preprocessing is an important step in ML that turns text data into a suitable format, which allows prediction and reproducibility (16). We opted a Scikit-learn (17) pre-built vectorizer using the Term Frequency—Inverse Document Frequency (TF-IDF) model approach (18). We also used a preprocessing function that covers the punctuation removal, the text normalization, and the tokenization. Due to language specificities, the diacritics were kept, and we used a French and a German dictionary to remove stopwords beforehand. Since some operations from ML algorithms involve randomization, we finally fixed the seed value to control the process and to be able to repeat it.

• Tokenization

We investigated different text splitting approaches to determine whether the ML models perform better on unigrams or on n-grams (19). To find the best thresholds, we experimented linearly different ranges per dataset based on words or character tokenizers. Such processing may have a significant impact on ML tasks, especially with free-text data where the context is crucial to understand the meaning of the sentence (*e.g.* with the negation in the phrases “*pas d’adénopathie abdomino-pelvienne*” (no abdomino-pelvic adenopathy), or with estimations “*majoration en taille*” (size augmentation) vs. “*diminution en taille*” (size decrease)).

• Cross-validation

To limit bias regarding the training/testing data sampling, we adopted the leave-one-out (LOO) cross-validation method (20, 21). Each item (a conclusion report) is used once as a test set (singleton) while the remaining documents form the training set. LOO may result in estimators with high variance in the case of high heterogeneity between samples. However, such a risk is limited by the nature of the processed documents compared to the generalization error (*i.e.,* a measure of how accurately an algorithm is able to predict outcome values for previously unseen data) often overestimated by 10-fold cross-validation.

• Classification strategies

We implemented seven approaches using the python package Scikit-learn: Linear Support Vector Machine (LinearSVM), Linear Stochastic Gradient Descent (LinearSGD), Gradient Boosting (GB), Logistic Regression (LR), Naive Bayes, Decision Tree and Random Forests.

• Hyperparameters-tuning

We focused on the optimization of the classification models *via* their hyperparameters tuning (22). Hyperparameters are specific settings used to control the learning process (*e.g.* learning rate in an SVM, number of branches in a decision tree, etc.) and thus have a direct impact on the performances of the models. As there is no way to determine in advance the best tuning of hyperparameters, this optimization must be set for any new predictive modeling task. Various strategies can be employed to find the right tuple of hyperparameters that yields an optimal learning. Regarding our experiments, we decided to perform an exhaustive search through a set of possible values. For each algorithm, we provided a rather large range of values for each hyperparameter, and a grid-search function automatically tested all the possible combinations to return the best solution.

### 2.5. Experimental designs

Our experiments were carried out on three fronts. First, designated as INSTITUTIONAL, our first batch of experiments compares seven classification strategies that already achieved state-of-the-art results on biomedical text-mining tasks. With the aim of providing each institution with an all-in-one annotation tool optimized for its own radiology reports, we evaluate the ML-based pipeline separately on the two datasets from the FRENCH corpus. On a first run, we tested the different models using their default parameters and with no tokenization of the text to create a baseline. Then, each model was optimized on successive runs following the pipeline described above.

Second, we conducted an experiment labeled INTEROPERABILITY wherein we assessed the performances of the two models trained on the INSTITUTIONAL task using a different dataset from the FRENCH corpus. The model trained on HUG dataset was tested on CHUV dataset, and *vice versa*. In other words, we examined to what extent the optimized models perform on new unseen data (*i.e.,* on external validation (23)). The goal of this batch of experiments was also to evaluate to what extent the data interoperability between different Swiss health centers can be considered.

Third, designated as LANGUAGE, we compared both FRENCH and GERMAN corpora to explore inter-language variations. With the aim of highlighting the peculiarities of Swiss languages, this last set of experiments was designed to showcase the issues that can be encountered when working with non-standardized textual data. Within the experimental framework, the models were individually trained and tested on each language, and the hyperparameters tailored to the particular linguistic context.

### 2.6. Evaluation metrics

To empirically select the best models, we compared their performances on basic settings according to standard ML metrics: accuracy and micro F1-score (24, 25). Accuracy is the ratio of the number of good predictions over the total number of predictions. Even if it is not the case on our datasets, this metric is not always appropriate to assess highly unbalanced classes distribution, therefore we also calculate the F1-score. F1 represents the harmonic mean between the precision and the recall (sensitivity) of the system.

Two additional metrics, namely the Matthews correlation coefficient (MCC) and the Cohen's Kappa, were computed to confirm the reliability of the systems depending on the type of classification. The Matthews correlation coefficient (MCC) is a complementary metric used in ML to measure the quality of a binary classification (26, 27). This coefficient is set on a range from −1 to +1 and performs even if the two classes are unbalanced: +1 represents a perfect prediction, 0 an average random prediction, and −1 indicates a total disagreement between predictions and observations. Besides, the Cohen's Kappa score is similar to IAA (f-score) since it also measures a level of agreement between different raters. However, the Cohen's Kappa is a statistical coefficient that integrates the probability of assigning the same labels by chance (28). It can be generally applied to ML classification tasks (using the true values and the predicted ones) to show to what extent a model is more successful than the random assignment of labels.

## 3. Results

The results in this section are presented in three parts, related to the experimental designs presented in the methodology (INSTITUTIONAL, INTEROPERABILITY, and LANGUAGE). The comprehensive results are reported in Supplementary Material “Comparison of the models”.

### 3.1. Institutional

On the FRENCH corpus, most classifiers tend to obtain very similar results on the classification of radiological documents. The Tables 2–5 display the results of the optimized ML-based algorithms on both CHUV and HUG datasets.

**Table 2:** Comparison of the ML performances on binary classification of the CHUV radiology reports.

Model	Linear SVM	Linear SGD	Gradient Boosting	Logistic Regression	Naive Bayes	Decision Tree	Random Forest
F1-score Non-progressive	0.88	0.87	0.90	0.88	0.86	0.87	0.89
F1-score Progressive	0.85	0.84	0.89	0.85	0.83	0.85	0.74
Accuracy	0.87	0.86	0.90	0.87	0.85	0.86	0.77
MCC	0.74	0.74	0.79	0.74	0.70	0.72	0.75

**Table 3:** Comparison of the ML performances on RECIST classification of the CHUV radiology reports.

Model	Linear SVM	Linear SGD	Gradient Boosting	Logistic Regression	Naive Bayes	Decision Tree	Random Forest
F1-score CR	0.67	0.73	0.75	0.73	0.75	0.42	0.47
F1-score PR	0.80	0.82	0.88	0.81	0.80	0.64	0.70
F1-score SD	0.62	0.65	0.86	0.71	0.61	0.80	0.72
F1-score PD	0.82	0.84	0.89	0.86	0.83	0.72	0.82
Accuracy	0.77	0.79	0.87	0.81	0.78	0.67	0.75
Cohen's Kappa	0.64	0.67	0.80	0.71	0.66	0.53	0.60

**Table 4:** Comparison of the ML performances on binary classification of the HUG radiology reports.

Model	Linear SVM	Linear SGD	Gradient Boosting	Logistic Regression	Naive Bayes	Decision Tree	Random Forest
F1-score Non-progressive	0.90	0.90	0.91	0.91	0.88	0.89	0.82
F1-score Progressive	0.80	0.80	0.85	0.86	0.76	0.82	0.52
Accuracy	0.87	0.87	0.88	0.89	0.84	0.86	0.74
MCC	0.71	0.71	0.76	0.77	0.65	0.71	0.42

**Table 5:** Comparison of the ML performances on RECIST classification of the HUG radiology reports.

Model	Linear SVM	Linear SGD	Gradient Boosting	Logistic Regression	Naive Bayes	Decision Tree	Random Forest
F1-score CR	0.75	0.79	0.82	0.73	0.76	0.90	0.85
F1-score PR	0.65	0.65	0.84	0.57	0.49	0.83	0.67
F1-score SD	0.66	0.83	0.81	0.67	0.60	0.81	0.77
F1-score PD	0.81	0.70	0.74	0.83	0.77	0.80	0.86
Accuracy	0.74	0.76	0.79	0.73	0.69	0.83	0.80
Cohen's Kappa	0.63	0.67	0.72	0.62	0.57	0.76	0.73

The award for the highest scores on both tasks in the CHUV dataset goes to the Gradient Boost model. In terms of binary classification, the system achieves an accuracy of 90% and F1-scores of 90% and 89% respectively for Progressive and Non-progressive. This represents an improvement of about 3% compared to the baseline and the MCC score of 79% indicates a high-quality classification performance.

Regarding the RECIST classification, the Gradient Boost shows an accuracy of 87% and F1-scores ranging from 75% (CR) to 89% (PD), steering an improvement of about 16% compared to the baseline. These F1-scores are relatively close to the IAA of 89%. Furthermore, the Cohen's Kappa value of 80% highlights the reliability of the model in this task.

On the HUG dataset, two different approaches stand out for each task. For binary classification (2-classes), the Logistic Regression method demonstrates strong performance, yielding an accuracy of 89%. The F1-scores for Progressive and Non-progressive predictions are 86% and 91% respectively. The optimized model shows an improvement of nearly 4% compared to the baseline, making it competitive with the inter-annotator agreement of 90%. The MCC score is 77%, further suggesting a robust classification quality.

Turning to the RECIST classification task, the Decision tree model yields good results. It achieves an accuracy of 83%, showcasing an improvement of about 16% when compared to the baseline. The F1-scores range from 80% (PD) to 90% (CR), which is close to the IAA of 94%. Additionally, the reported Cohen's Kappa coefficient is 76%, indicating the reliability of this model.

### 3.2. Interoperability

**Figure 1:**
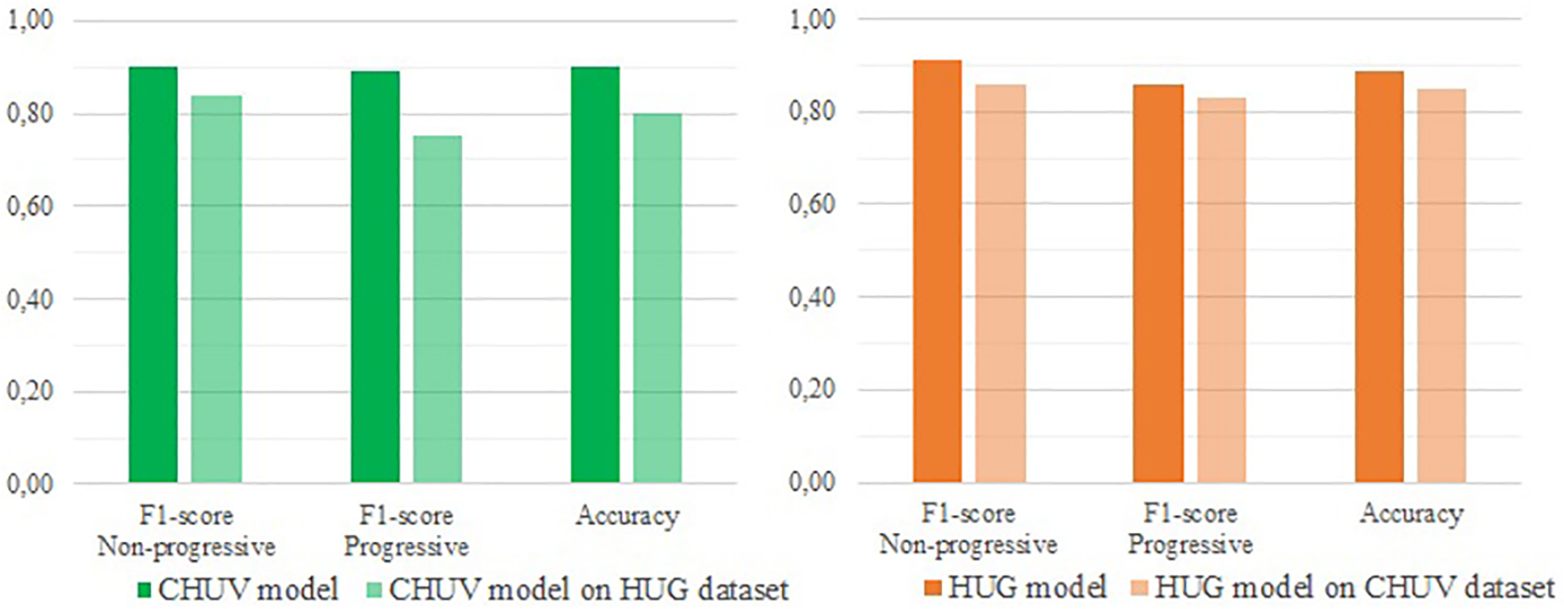
External validation of both CHUV and HUG optimized models (respectively GB and LR) regarding the 2-classes (progressive/Non-progressive) classification.

**Figure 2:**
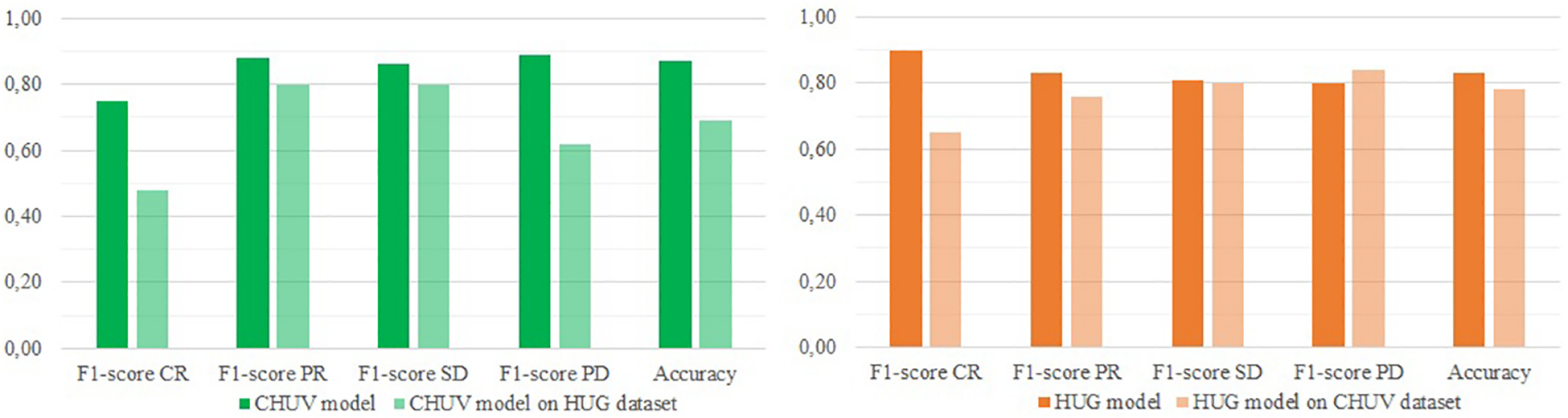
External validation of both CHUV and HUG optimized models (respectively GB and decision tree) regarding the 4-classes RECIST classification.

The goal of the second batch of experiment was to test the performance of the optimized models described above when working on new data. The Figures 1 and 2 present the results of this external validation.

On the binary classification task, the model trained on the CHUV (a Gradient Boost) data yields an accuracy of 80%, with F1-scores of 75% (Progressive) and 84% (Non-progressive), and a MCC of 59%. In comparison, the model suited to the 4-classes classification (also a Gradient Boost) gets an accuracy of 69%, with F1-scores ranging from 48% (CR) to 80% (SD) and a Cohen's Kappa of 56%.

The models trained on the HUG data reach slightly higher scores. On the binary classification, the model (a Logistic Regression) achieves an accuracy of 85%, with F1-scores of 83% (Progressive) and 86% (Non-progressive), and a MCC of 70%. On the 4-classes classification task, the Decision Tree model obtains an accuracy of 78%, with F1-scores from 65% (CR) to 84% (PD) and a Cohen's Kappa of 69%.

### 3.3. Language

**Figure 3:**
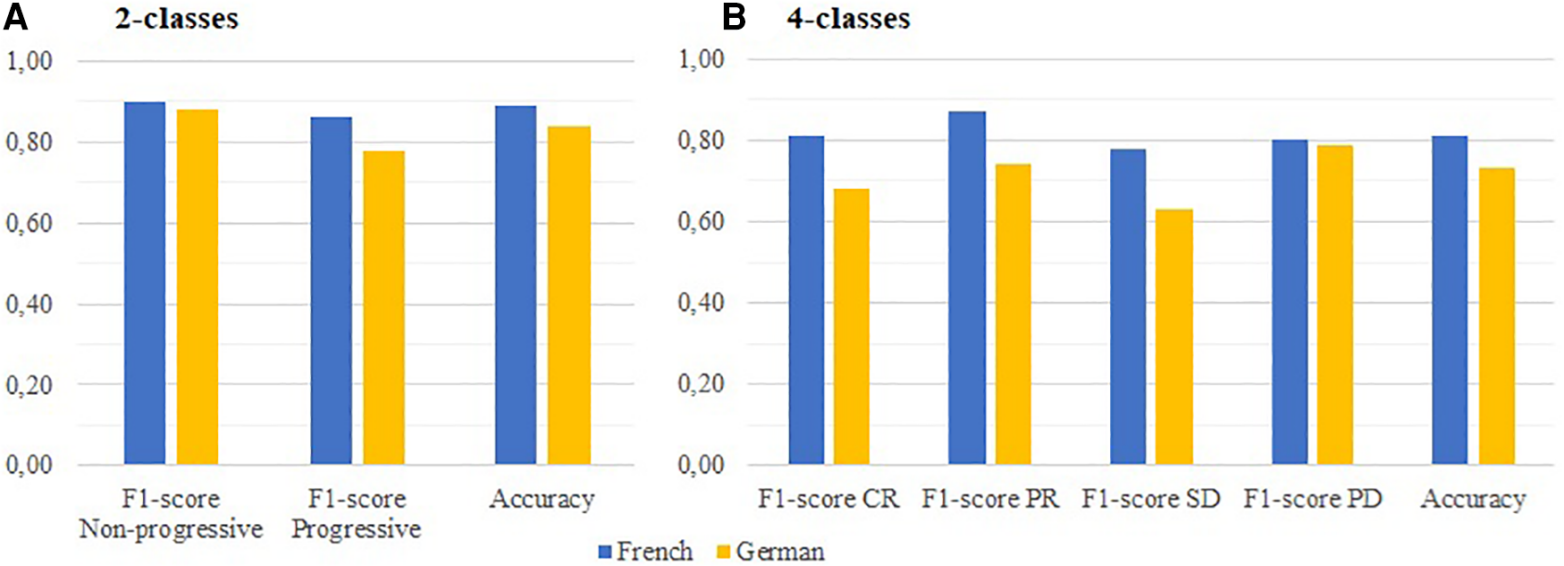
Performances of the top ML-based strategies on FRENCH and GERMAN corpora, regarding the: (**A**) Progressive/Non-progressive classification (LR vs. LinearSGD); (**B**) 4-classes RECIST classification (LR vs. GB).

In this section, we present the results of ML-based experiments conducted on the FRENCH and GERMAN corpora to examine inter-language differences. The cross-validation approach exploits the complete corpora, including the institutional datasets. Figure 3 illustrates the performance of the best models trained specifically on French or German conclusions.

On the German documents, the Logistic Regression model demonstrates good results for both tasks. On the binary classification task, the model achieves an accuracy of 84%, F1-scores of 78% (Progressive) and 88% (Non-progressive), and a MCC of 70%. On the 4-classes classification task, the accuracy drops to 73%, while the F1-scores range from 63% (SD) to 79% (PD). The Cohen's Kappa is reported as 69%.

On the French documents, the LinearSGD achieves scores comparable to the INSTITUTIONAL batch of experiments for the binary classification. It reaches an accuracy of 89%, F1-scores of 89% (Progressive) and 90% (Non-progressive), and a MCC of 76%. Looking at the RECIST classification, two models (Gradient Boost and Logistic Regression) prove to be suitable for the task with equal accuracies of 81% and Cohen's Kappa of 73%. However, the Gradient Boost shows slightly better performance with narrower F1-scores ranging from 78% (SD) to 87% (PR).

## 4. Discussion

The findings of this study indicate that automating the labeling of radiological reports to infer progression of cancers can achieve a reliable classification rate, approaching human judgement as it is assessed with the IAA. However, the data capture policy of every healthcare institution and the quality of the clinical narratives may influence the quality of the automation.

In the subsequent sections, we have examined the primary factors contributing to misclassifications. Yet, the strict annotation guidelines and the document selection procedure described in the methods have efficiently mitigated the risk of “data shift”, “class definition” and “class imbalance”.

### 4.1. Institutional

On the first batch of experiments, considering the relatively good results of the top performing models, there is little room for improvement using traditional ML approaches. With an MCC of 77% and 79% respectively for HUG and CHUV systems, these scores indicate that the binary predictors were able to correctly predict most of the positive data instances as well as the negative data instances. On the 4-classes classification task, the Cohen's Kappa of 76% obtained by the HUG model indicates a strong agreement between the inferences from the Logistic Regression and the gold standard used for the evaluation. Similarly, the Cohen's Kappa of 80% on the CHUV data suggests that this model is very reliable. Yet there are questions about how the misclassifications occurred (see Supplementary Materials “Confusion matrices” for a report on inaccuracies).

**Figure 4:**
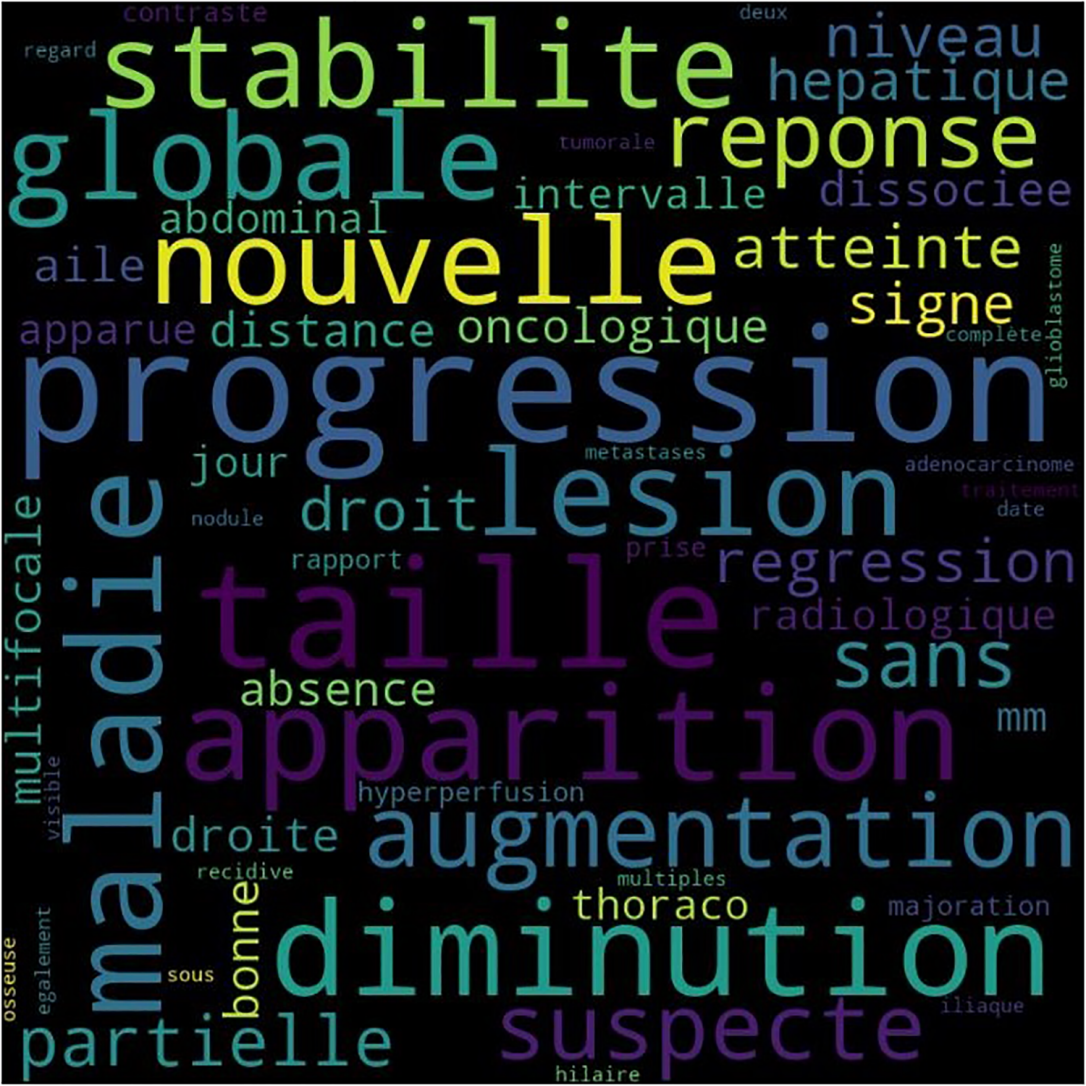
Word cloud displaying the top 50 features in French documents.

The presence of *hapax* (*i.e.,* word or expression that occurs only once in the whole dataset) is responsible for a significant number of misclassifications, especially in short texts where the inference is biased by the few features the system has already detected in its training set. Another predominant source of misclassification stems from the simultaneous presence of high-weighted features in a given document, typically observed in cases involving dissociated responses (see example in Box 1). Figure 4 presents the tokens of influence that can be found in the French radiology reports.

Box 1 **Extract from a French note presenting a dissociated response that results in a misclassification. Here, the ML-based algorithm estimates a CR instead of a PD, with a confidence score of 55%.**

"*Réponse dissociée de la maladie avec progression du nodule axillaire gauche et disparition des foyers hépatique et pancréatique*” (Dissociated response of the disease with progression of the left axillary nodule and disappearance of the hepatic and pancreatic foci)

### 4.2. Interoperability

On the cross-validation experiments, we observed that the performances of all the investigated models have decreased. The variability among authors' writing styles is the principal cause of misclassification at document level. Thus, some reports extracted from the hospitals' databases do not seem to comply with the institutional templates (*e.g.* several reports do not include a conclusion section). In some preselected documents, we observed that the absence of a standard way to formulate the radiological observations entails more original features for the learning models; especially considering the German language, which combines concepts such as “*Glioblastom-metasasen-resektions-bereich*” (area of glioblastoma metastasis resection). On top of that, the expression of some degree of confidence (and *doxa*) might also affect the learning of the classifier, for instance in “*metastasensuspekt*” (suspicious for metastasis) vs. “*metastasen*” (metastasis).

As it is known that ML strategies are data dependent and that overfitting phenomena are a possible limitation, we have sought to limit the bias in comparing the models by focusing on the conclusion of the radiology reports. However, when performing tuned models on documents from different institutions, the two systems have shown a substantial fall in their scores. Beyond the current lack of interoperability between data sources, such an outcome is related to the variability in the training data. A drop in the models' performances on external validation can be interpreted as overfitting (29), although limited in our case. Instead of learning how to generalize from features, the models are moderately memorizing non-predicting items.

### 4.3. Language

On the third batch of experiments, one of the main issues concerns the words that implied some form of negation. For example, in French, these markers can be words from different parts of speech: adverbs (such as “*pas (de)*” (not) or “*jamais*” (never)), nouns (such as “*absence*” (absence)), adjectives (such as “*aucun*” (none)) or others. Yet, in German the negative forms appear to be particularly problematic for the classification of reports (30). In some cases, the negation is close to the subject and a simple n-gram tokenizer can handle it (*e.g.,* in “*Kein Hinweis auf eine lymphogene oder hämatogene Metastasierung.*” (No hint on lymphogenic or hematogenic spread of metastases)). Notwithstanding all this, our language agnostic approach could potentially be affected by such phenomena, but our observations suggest that the impact was null or marginal on our results.

And finally, beyond linguistics, a collateral problem related to human practices emerged from the supplied data. Indeed, by focusing on German documents it appears that a few sentences also integrate English and Latin words. Even if it does not block the human understanding of the report, this may lead to the genesis of new learning features. In addition to a few peculiarities from Swiss German, we also observed a slightly greater number of typographical errors in the examined German reports than in the French ones. Depending on the typographical error, they may impact the learning features as well as any words in another language. Yet, it seems that the detected typographical errors have a marginal effect on the classification since the systems handle most of them with a tokenizer and a simple stemming pre-processing of the texts (we tested the generic function from Python NLTK library). More elaborated methods to correct spelling corruptions seemed therefore unnecessary as explored in the literature (31).

### 4.4. Future direction

Despite achieving good results, ML methods have inherent limitations in their quantitative interpretation of the information rendering them susceptible to overfitting. The concern is particularly pronounced when working with limited datasets. In contrast, pre-trained language models, built upon extensive training on large corpora, offer a more robust framework to address overfitting. Indeed, PLMs capitalize on the ability to extract unsupervised patterns from larger datasets, which can subsequently be fine-tuned or customized using a smaller set of annotated samples (29). In addition to overfitting, some traditional machine learning approaches may struggle to discriminate between classes when documents contain features from multiple categories, as corroborated by our error analysis. Pre-trained language models leverage their extensive training and possess a deep understanding of language semantics and context, some of them being trained specifically on data from healthcare centers (32, 33).

To extend the scope of our study, we therefore experimented CamemBERT, a multi-layer bidirectional Transformer designed as a French adaptation of the ROBERTA model (34). Pretrained on highly variable datasets, and with little effort on optimization, the CamemBERT model immediately showed results very similar to our optimal ML-based strategies on the INSTITUTIONAL set of experiments (CamemBERT F1-scores of 90% on the binary classification and ranging from 80% to 90% on the 4-classes classification). It also appears that a combination of the two approaches could be interesting to explore in future work.

## 5. Conclusion

Our study examined different traditional ML-based models to support the classification of free-text radiological documents. The strategy relies on expert manual annotations and on an optimization relating to each dataset in order to infer on novel documents. The resulting systems show performances that approach the inter-annotator agreement (up to 91%) and are particularly well suited to the binary classification task with top F1-scores that reach 90% to 91% respectively for “Progressive” and “Non-progressive” predictions. Moreover, the results established on the 4-classes classification are also satisfying with an average F1-score of 85% reached at best. Yet, when evaluating the capacity of the models to generalize on new data, the performances of both CHUV and HUG classifiers have respectively dropped by about 11% and 4% on the 2-classes classification task (−20% and −9% on the 4-classes classification).

By analyzing the misclassification instances, different issues have emerged. Some of these issues are dataset dependent while others typically stem from the data variability in radiology reports. For instance, it appears that learning from negations in documents written in German is not quite simple and, given the limited set of training/tuning data, additional NLP preprocessing may help. Pre-trained language models can here provide a sound alternative to such data labeling issues. We also highlighted some singletons that currently represent a bottleneck to evaluate the models, as well as the “dissociated response” cases which remain ambiguous for both the human and the machine. However, until the variance/bias threshold is not reached, all these classification models would certainly benefit from larger datasets.

## Data Availability

The datasets presented in this article are not readily available because of patient confidentiality. Requests to access these datasets should be directed to Luc Mottin, luc.mottin@hesge.ch.
