# Supplementary material for: Multilingual RECIST classification of radiology reports using supervised learning
Source: Front Digit Health. 2023 Jun 14;5:1195017. doi: 10.3389/fdgth.2023.1195017 (PMC10303934; doi:10.3389/fdgth.2023.1195017)
Supplement: Supplementary file 1 [file Datasheet1.docx]

Supplementary Material

**Multilingual RECIST classification of radiology reports using supervised learning**

Luc Mottin^1,2*^, Jean-Philippe Goldman^3^, Christoph Jäggli^4^, Rita Achermann^5^, Julien Gobeill^1,2^, Julien Knafou^1,2^, Julien Ehrsam^6^, Alexandre Wicky^7^, Camille L. Gérard^7^, Tanja Schwenk^8^, Mélinda Charrier^3^, Petros Tsantoulis^3,6^, Christian Lovis^3,6^, Alexander Leichtle^4^, Michael K. Kiessling^9^, Olivier Michielin^7^, Sylvain Pradervand^7^, Vasiliki Foufi^3^, Patrick Ruch^1,2^

^1^ HES-SO\HEG Genève, Information Sciences, Geneva, Switzerland

^2^ SIB Text Mining, Swiss Institute of Bioinformatics, Geneva, Switzerland

^3^ Geneva University Hospitals, Geneva, Switzerland

^4^ Inselspital – Bern University Hospital and University of Bern, Bern, Switzerland

^5^ Universitätsspital Basel, Basel, Switzerland

^6^ University of Geneva, Geneva, Switzerland

^7^ Centre Hospitalier Universitaire Vaudois – CHUV, Precision Oncology Center, Oncology Department, Lausanne, Switzerland

^8^ Kantonsspital Aarau, Department of Oncology, Aarau, Switzerland

^9^ UniversitätsSpital Zürich, Zurich, Switzerland

*** Correspondence:**Luc Mottin
[luc.mottin@hesge.ch](mailto:luc.mottin@hesge.ch)

# Treatment Response Guidelines (2019)

Objective: Extract SPO treatment response variables from radiology reports for the text mining tasks

**Report selection**

**Inclusion criteria reports:**

- 6 major tumor types:
  - Breast (C50, all subclasses)
  - Gastro-intestinal (C18, C19, C20, all subclasses)
  - Lung (C34, all subclasses)
  - Prostate (C61, all subclasses)
  - Melanoma (C43, all subclasses)
  - Glioblastoma (no ICD-10 code Glioblastoma, Malignant neoplasm of brain C71)
- CT, MRI and PET radiology reports from cancer patients from exams performed between 2 and 4 months after administration of every and any drug treatment (chemotherapy, hormonotherapy, targeted therapy, immunotherapy) for gastro-intestinal, lung, melanoma and glioblastoma. For breast and prostate, only patients treated by chemotherapy will be selected.
- Dosage of chemotherapy is not relevant.
- Not restricted to metastatic patients (such a restriction complicates report selection)
- To identify the reports, the administration date of the chemotherapy is taken independent of whether it is the start of the therapy or not.

**Exclusion criteria reports:**

- Reports from patients with surgery or radiation therapy will not be assessed if no drug treatment is applied.
- Treatment outcome from reports with RECIST information will be extracted by parsing and not text mining procedure (‘author traceable statement’ is extracted by text mining).
- Reports with no clearly identifiable ‘conclusion’ section will not be assessed.
- Exams that are performed for other reasons than treatment response assessment: surgery planning, etc. Of note, reports should not be selected by a manual procedure. Exclusion criteria are hospital dependent. Examples: exclude reports ordered by surgical department.

**Labeling**

- Limit text mining classification to the conclusion section.
- Value set of labels according to RECIST: complete response (CR), partial response (PR), stable disease (SD), progression (PD), unknown.
- If there is *New lesion* somewhere, then the label “Progression” is chosen.
- If no evidence of tumor: always complete response.
- A subcategory ‘Dissociated response’=[yes/no] is added in order to take into account the reporting of lesions responding differently to the treatment. For example, you can have lung metastasis that completely disappear, but a new lesion in the liver. In this case, the label will be ‘PD’ with ‘Dissociated response’=yes.
- An attribute ‘Low confidence’=[yes/no, default no] is added for cases where the author speculates about the response. The assumption is that the exam will be repeated. Example: Could be an infection or tumor progression.
- If the author of the report doesn’t state any conclusion the label is set to ‘unknown’.
- If the results of the exam are compared with multiple exams from the past, follow RECIST rules that state that comparisons be made against the nadir, *i.e.* the best exam since treatment start. So, a patient could have, sequentially: SD, PR, PR, SD, PD. In that case, the best obtained response is PR. This is documented in https://ctep.cancer.gov/protocolDevelopment/docs/recist_guideline.pdf section 4.3.1.
- Each report will be assessed on its own, one label per report. A patient can have more than one label at a specific time point if multiple exams are performed. Example: brain metastasis assessed with MRI for patients with lung cancer (CT thorax).
- If the automatic report selection is not specific enough, *e.g.* contains reports that are not relevant for treatment assessment, an additional label <report not relevant> has to be considered. A classifier can then be trained to identify relevant reports.

**Limitations**

- There is no guarantee on the performance of the classifiers.
- There may be discrepancies between ‘author statement’ and RECIST evaluation.
- The classifier is based on a single report. The result may therefore differ from the oncologist conclusion in discharge letter.
- No evaluation of surgery, radiotherapy, interventional radiology or any other non-drug treatment.
- Provide each prediction with a probability estimate - also performed by the annotator.
- The guidelines do not define any comparison point to establish the treatment response. Such a temporal point would be needed, for instance, to compute progression-free survival (PFS).

# Comparison of the models

## INTEROPERABILITY dataset

Comparison of the ML performances on French data by crossing the datasets, for a) 2-classes classification

| Model | CHUV | HUG |
| --- | --- | --- |
| F1 score Non-progressive | 0.84 | 0.86 |
| F1-score Progressive | 0.75 | 0.83 |
| Accuracy | 0.80 | 0.85 |
| MCC | 0.59 | 0.70 |

b) 4-classes classification

| Model | CHUV | HUG |
| --- | --- | --- |
| F1-score CR | 0.48 | 0.65 |
| F1-score PR | 0.80 | 0.76 |
| F1-score SD | 0.80 | 0.80 |
| F1-score PD | 0.62 | 0.84 |
| Accuracy | 0.69 | 0.78 |
| Cohen's Kappa | 0.56 | 0.69 |

## LANGUAGE dataset: FRENCH

Comparison of the ML performances on binary classification of the combination of French radiology reports.

| Model | Linear SVM | Linear SGD | Gradient Boosting | Logistic Regression | Naive Bayes | Decision Tree | Random Forest |
| --- | --- | --- | --- | --- | --- | --- | --- |
| F1-score Non-progressive | 0.90 | 0.90 | 0.88 | 0.89 | 0.87 | 0.87 | 0.89 |
| F1-score Progressive | 0.85 | 0.86 | 0.84 | 0.85 | 0.79 | 0.81 | 0.79 |
| Accuracy | 0.88 | 0.89 | 0.86 | 0.87 | 0.84 | 0.84 | 0.85 |
| MCC | 0.75 | 0.76 | 0.72 | 0.74 | 0.66 | 0.67 | 0.69 |

Comparison of the ML performances on RECIST classification of the combination of French radiology reports.

| Model | Linear SVM | Linear SGD | Gradient Boosting | Logistic Regression | Naive Bayes | Decision Tree | Random Forest |
| --- | --- | --- | --- | --- | --- | --- | --- |
| F1-score CR | 0.79 | 0.85 | 0.81 | 0. 83 | 0.84 | 0.78 | 0.82 |
| F1-score PR | 0.74 | 0.75 | 0.87 | 0.74 | 0.72 | 0.79 | 0.74 |
| F1-score SD | 0.70 | 0.70 | 0.78 | 0.74 | 0.59 | 0.70 | 0.77 |
| F1-score PD | 0.84 | 0.85 | 0.80 | 0.86 | 0.84 | 0.78 | 0.84 |
| Accuracy | 0.79 | 0.80 | 0.81 | 0.81 | 0.77 | 0.76 | 0.81 |
| Cohen’s Kappa | 0.69 | 0.71 | 0.73 | 0.73 | 0.67 | 0.67 | 0.72 |

# Confusion matrices of leading strategies on 4-classes RECIST classification of French reports


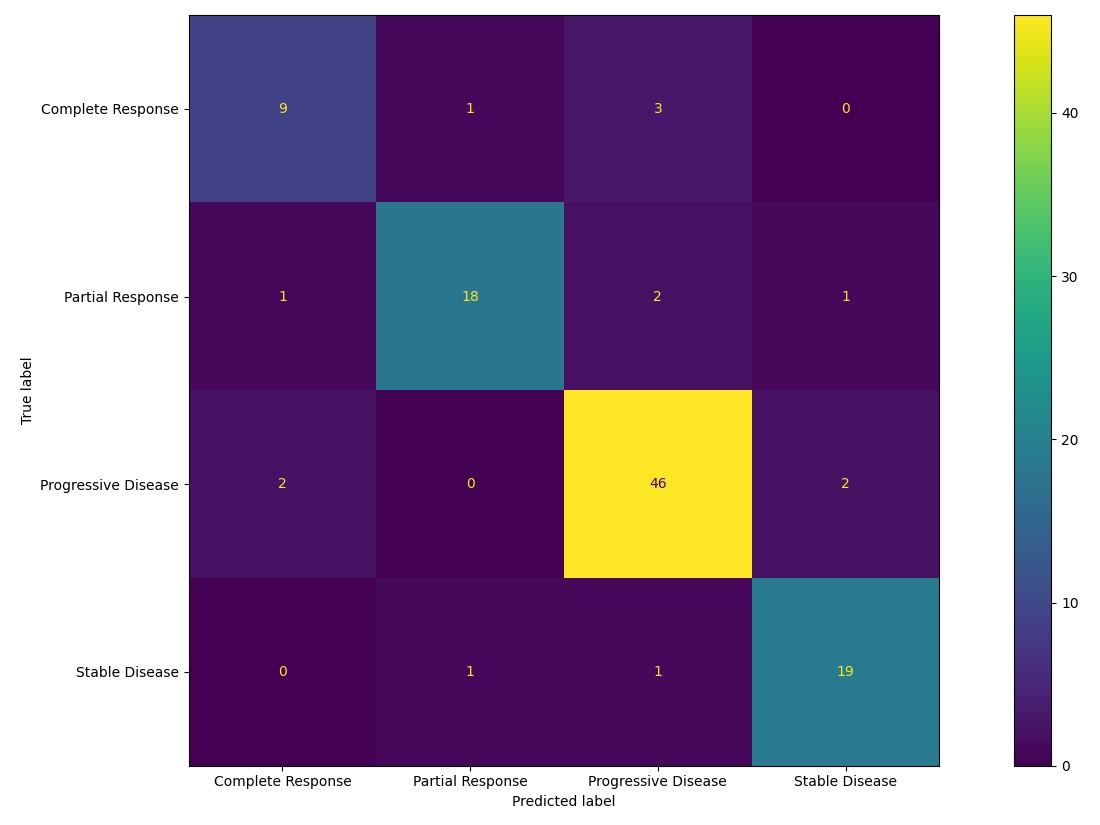


**Supplementary Figure 1.** Confusion matrix of 4-classes classification using Gradient Boost optimized model on CHUV dataset.


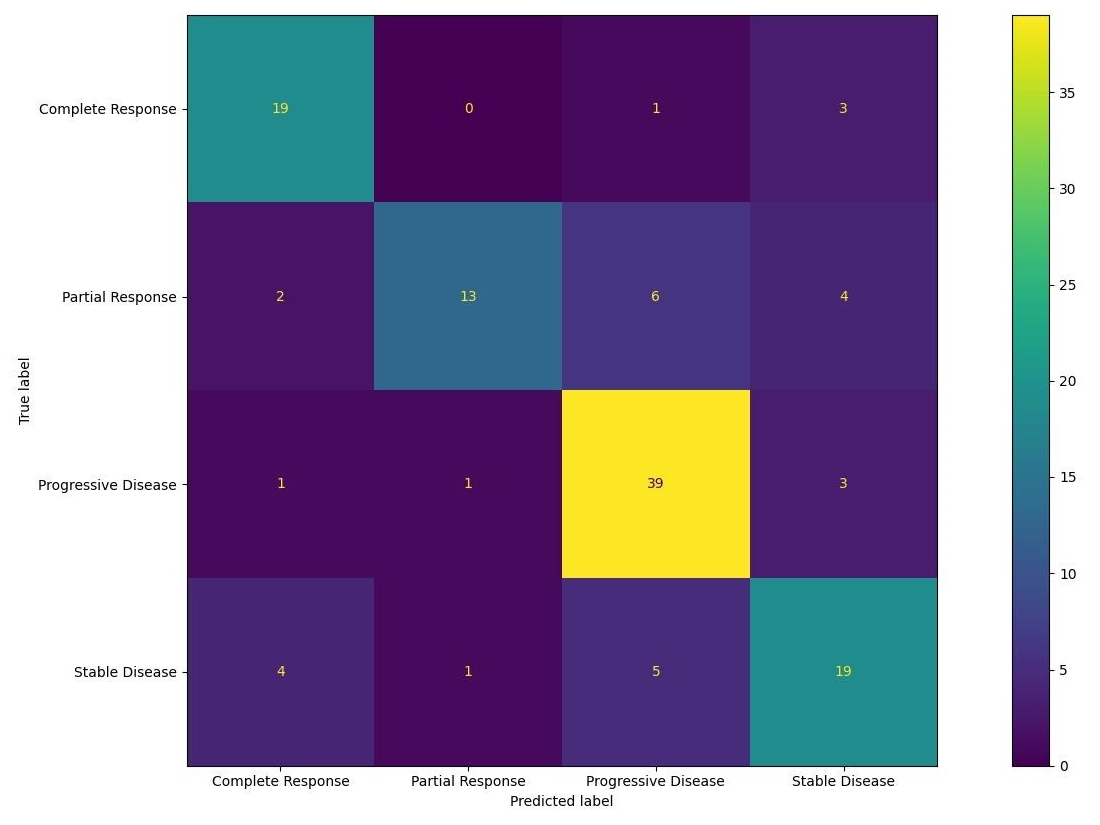


**Supplementary Figure 2.** Confusion matrix of 4-classes classification using Decision Tree optimized model on HUG dataset.

# Inter-annotator agreement evaluation

## Inter-annotator agreement per disease on CHUV dataset (2 annotators)

|  | BREAST | BRAIN | LUNG | MELANOMA | PROSTATE | GI | ALL |
| --- | --- | --- | --- | --- | --- | --- | --- |
| Complete Response | 3 | 0 | 3 | 1 | 0 | 6 | 13 |
| Partial Response | 5 | 1 | 6 | 3 | 2 | 5 | 22 |
| Stable Disease | 5 | 8 | 3 | 0 | 3 | 2 | 21 |
| Progressive Disease | 4 | 10 | 5 | 14 | 12 | 5 | 50 |
| Total (%) | 85% | 95% | 85% | 90% | 85% | 90% | 88,3% |

## Inter-annotator agreement on CHUV dataset (3 annotators^[[1]](#footnote-1)^)

| A | B | F1-score | Agreement (n/120) | Agreement (%) |
| --- | --- | --- | --- | --- |
| Annot1 CHUV | Annot2 CHUV | 0.93 | 106 | 88.3% |
| Annot1 CHUV | Annot1 HUG | 0.82 | 99 | 82.5% |
| Annot2 CHUV | Annot1 HUG | 0.83 | 99 | 82.5% |
| Gold | Annot1 CHUV | 0.90 | 106 | 88.3% |
| Gold | Annot2 CHUV | 0.90 | 108 | 90.0% |
| Gold | Annot1 HUG | 0.92 | 110 | 91.7% |

## Inter-annotator agreement on HUG dataset (3 annotators)

| A | B | F1-score | Agreement (n/122) | Agreement (%) |
| --- | --- | --- | --- | --- |
| Annot1 HUG | Annot2 HUG | 0.91 | 113 | 92.6% |
| Annot1 HUG | Annot3 HUG | 0.79 | 98 | 80.3% |
| Annot2 HUG | Annot3 HUG | 0.76 | 96 | 78.7% |
| Gold | Annot1 HUG | 0.90 | 110 | 90.1% |
| Gold | Annot2 HUG | 0.87 | 109 | 89.3% |
| Gold | Annot3 HUG | 0.89 | 107 | 87.7% |

1. a single expert annotator solved annotation disagreements, yielding to a gold-standard reference corpus [↑](#footnote-ref-1)
